# A Nine-Year-Old Child With Metastatic Pancreatic Adenocarcinoma

**DOI:** 10.7759/cureus.60670

**Published:** 2024-05-20

**Authors:** Katelin Magnan, Linford Williams, Qian Wang, Julia Meade

**Affiliations:** 1 Pediatrics, UPMC (University of Pittsburgh Medical Center) Children's Hospital of Pittsburgh, Pittsburgh, USA; 2 Medical Genetics, UPMC (University of Pittsburgh Medical Center) Children's Hospital of Pittsburgh, Pittsburgh, USA; 3 Department of Pathology, Children's Hospital of Pittsburgh, Pittsburgh, USA; 4 Pediatric Oncology, UPMC (University of Pittsburgh Medical Center), Pittsburgh, USA

**Keywords:** chemotherapy for pdac and biliary tract carcinoma, cancer-genetics, rare case report, survival of pediatric cancer, pancreatic adenocarcinoma treatment

## Abstract

Pancreatic ductal adenocarcinoma is exceedingly rare in children. Here, we report the case of a nine-year-old boy diagnosed with pancreatic ductal adenocarcinoma. The patient was treated per the National Comprehensive Cancer Network^®^ (NCCN^®^) guidelines for adults with pancreatic cancer. Though the patient had multiple episodes of progression, the patient has remained alive with the disease 18 months after the initial diagnosis.

## Introduction

Pancreatic cancer is a relatively rare malignancy in adults with an annual incidence of around ~66,000 new cases per year and a median age at diagnosis of 71 [1,2]. Pancreatic ductal adenocarcinoma (PDAC) accounts for the majority (90%) of pancreatic neoplasms in adults [2]. Unfortunately, PDAC carries a very poor prognosis, with an overall survival rate of less than 10% in adults [3,4]. Patients often present with either locally advanced or metastatic disease.

PDAC is exceedingly rare in adolescents and young adults, with 0.3% of total cases occurring in patients younger than 40 [5]. Reports of PDAC are essentially unheard of in children less than 10 years old. In younger patients, PDAC is more commonly associated with genetic factors, and several cancer predisposition syndromes have been implicated [5-7]. There are currently no evidence-based treatment guidelines for the management of PDAC in pediatric patients. Care of a child with PDAC requires extrapolation of existing adult cancer treatment recommendations to a pediatric population.

We report here a case of a rare diagnosis of pancreatic ductal adenocarcinoma in a nine-year-old boy. This patient was found to have a variant of uncertain significance (VUS) in the *FANCC* gene. He was treated according to adult treatment recommendations as per the adult National Comprehensive Cancer Network^®^ (NCCN^®^) PDAC guidelines. At the time of this report (18 months after diagnosis), he is still alive with progressive disease.

This article was previously posted to the ResearchGate preprint server on January 10, 2024.

## Case presentation

A nine-year-old boy presented with a three-week history of progressively worsening abdominal pain, vomiting, and diarrhea. The child had lost 13 pounds over the same timeframe. One day prior to the presentation, he developed jaundice and pruritus. He endorsed a darkening of his urine and pale stools over the past 24 hours as well. On presentation, he was afebrile with normal vital signs (temperature 36.2 degrees Celsius, heart rate 100, blood pressure 110/70, respiratory rate 20, oxygen saturation 97% on room air). The initial physical exam was notable for jaundice and scleral icterus as well as tenderness to palpation of the right upper quadrant, without appreciated masses or hepatosplenomegaly.

Laboratory workup was notable for direct hyperbilirubinemia (total bilirubin level 10.9 mg/dL, direct bilirubin level 6.6 mg/dL). gamma-glutamyl transpeptidase (GGTP) was elevated to 690 IU/L and lipase was elevated to 1225 IU/L. He also had transaminitis with alanine transaminase (ALT) 552 IU/L and aspartate aminotransferase (AST) 169 IU/L. A complete blood count revealed normal hemoglobin and white blood cells. Coagulation studies were also normal. Cancer antigen 19-9 (CA 19-9) was elevated at 162 U/mL (Table 1).

**Table 1:** Initial laboratory values of the patient PTT: partial thromboplastin time

Lab	Patient Results	Reference Range
Sodium	136 mmol/L	136- 146 mmol/L
Potassium	4.0 mmol/L	3.4- 4.7 mmol/L
Chloride	19.0 mmol/L	21- 31 mmol/L
Urea Nitrogen	5.0 mg/dL	6- 18 mg/dL
Creatinine	0.54 mg/dL	0.3- 0.6 mg/dL
Glucose	84 mg/dL	70- 126 mg/dL
Calcium	9.5 mg/dL	8.8- 10.3 mg/dL
Magnesium	2.0 mg/dL	1.6- 2.2 mg/dL
Phosphorus	4.7 mg/dL	4- 5.7 mg/dL
Albumin	3.6 g/dL	3.9- 4.9 g/dL
Total Bilirubin	10.9 mg/dL	0- 0.7 mg/dL
Indirect Bilirubin	4.3 mg/dL	0.2- 0.7 mg/dL
Direct Bilirubin	6.6 mg/dL	0- 0.2 mg/dL
Alanine Aminotransferase	552 IU/L	0- 32 IU/L
Aspartate Aminotransferase	169 IU/L	25- 55 IU/L
Alkaline Phosphatase	690 IU/L	142- 336 IU/L
Gamma-Glutamyl Transferase	212 IU/L	0- 45 IU/L
Lipase	1225 IU/L	73- 393
Cancer Antigen 19-9	162 U/mL	0- 34 U/mL
White Blood Cells	8,300/µL	4,500- 14,500/µL
Hemoglobin	13.0 g/dL	11.5- 15.5 g/dL
Platelets	291,000/µL	156,000- 369,000/µL
Prothrombin Time	13.1 seconds	11.7- 15.1 seconds
Activated PTT	29 seconds	31.8- 43.7 seconds
International Normalized Ratio	1.0	0.9- 1.2

**Figure 1:**
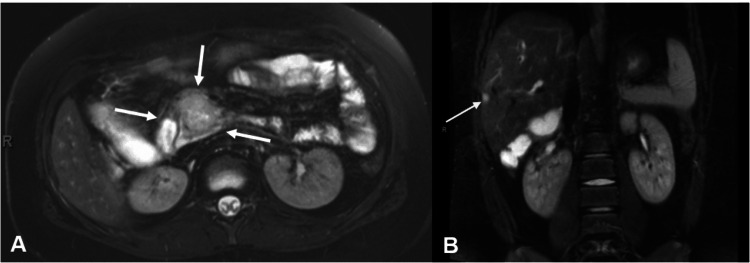
MRI of the abdomen with contrast demonstrates a pancreatic head mass (A) and solitary metastatic lesion to the liver (B) MRI: magnetic resonance image

Imaging was obtained, with abdominal ultrasound showing intrahepatic and extrahepatic biliary and pancreatic dilatation, concerning for obstruction. Magnetic resonance imaging (MRI) of the abdomen with contrast revealed a 3.3 x 2.7 x 3.4 cm obstructive abnormality in the uncinate process of the pancreas with extra- and intrahepatic dilation of the bile ducts in the liver, as well as a 1 cm enhancing lesion in the liver (Figure 1).

**Figure 2:**
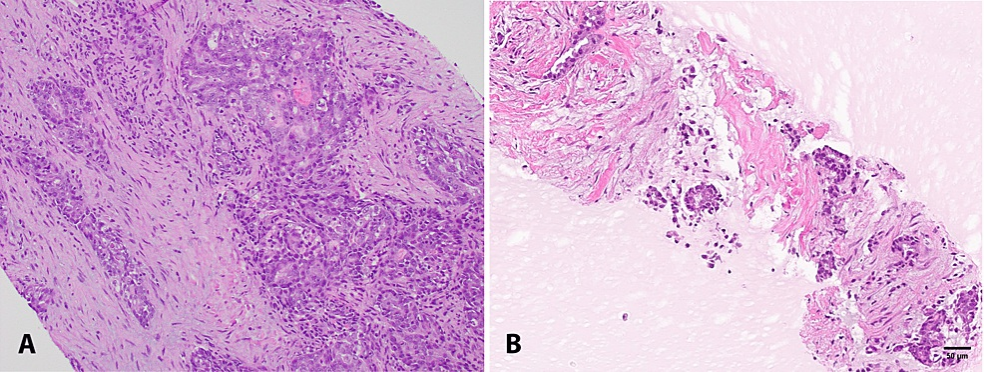
Pathology demonstrates pancreatic ductal adenocarcinoma (A) Core biopsy from the liver shows an adenocarcinoma with a glandular and cribriform pattern. (B) SharkCore endoscopic ultrasound (EUS)-guided fine-needle biopsy from the pancreas head shows an infiltrating tumor with similar morphological features as (A). Scale bar=50 um

Extensive pathology review demonstrated atypical epithelial proliferation forming mucin-producing irregular and anastomosing glands (Figure 2). Neoplastic cells were heterogeneously CK7 positive and beta-catenin membrane positive. SMAD4 was retained, and PAX8 was also weakly positive. Neoplastic cells were CK20, BCL10, synaptophysin, trypsin, CDX2, TTF-1, and SALL4 negative. The microarray of the tumor was negative for copy number abnormalities, DNA mutations, and gene fusions. The tumor was microsatellite stable with a tumor mutation burden of 3.4 mutations/Mb.

**Figure 3:**
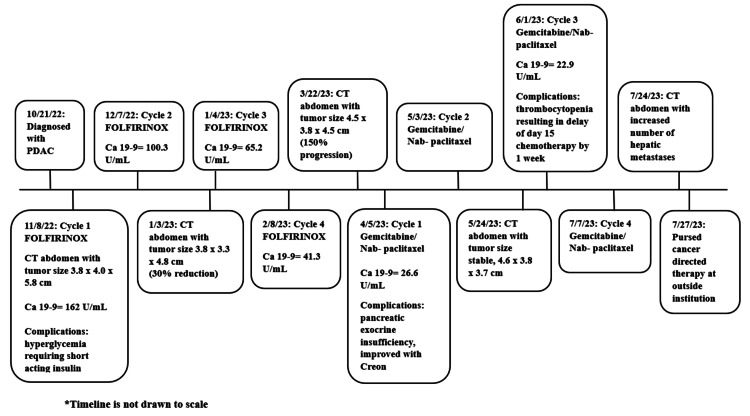
Timeline of patient's clinical course PDAC = pancreatic ductal adenocarcinoma; FOLFIRINOX = folic acid, fluorouracil, irinotecan, and oxaliplatin; Ca 19-9 = cancer antigen 19-9; CT = computed tomography

The child was started on chemotherapy with FOLFIRINOX (folic acid, fluorouracil, irinotecan, and oxaliplatin) as per the NCCN treatment guidelines and experienced a 30% reduction in tumor volume after two cycles with a biochemical response (CA 19-9 decreased from 162 U/mL pre-chemo to 65.2 U/mL). However, after cycle 4 of FOLFIRINOX, he was noted to have tumor progression of 150% on imaging. His regiment was changed to gemcitabine and nab-paclitaxel. He had one treatment delay during cycle 3 of gemcitabine/nab-paclitaxel secondary to thrombocytopenia. During this part of his treatment, he had one ED visit for a viral syndrome, but no concerns on evaluation for bacterial infection or sepsis. Unfortunately, he had further progression after cycle 4 of gemcitabine/nab-paclitaxel with an increased number of hepatic metastases and a slight increase in tumor size. The patient then pursued further cancer-directed therapy outside of our institution. Now 18 months out from initial diagnosis, the child is alive with progressive disease (Figure 3).

**Figure 4:**
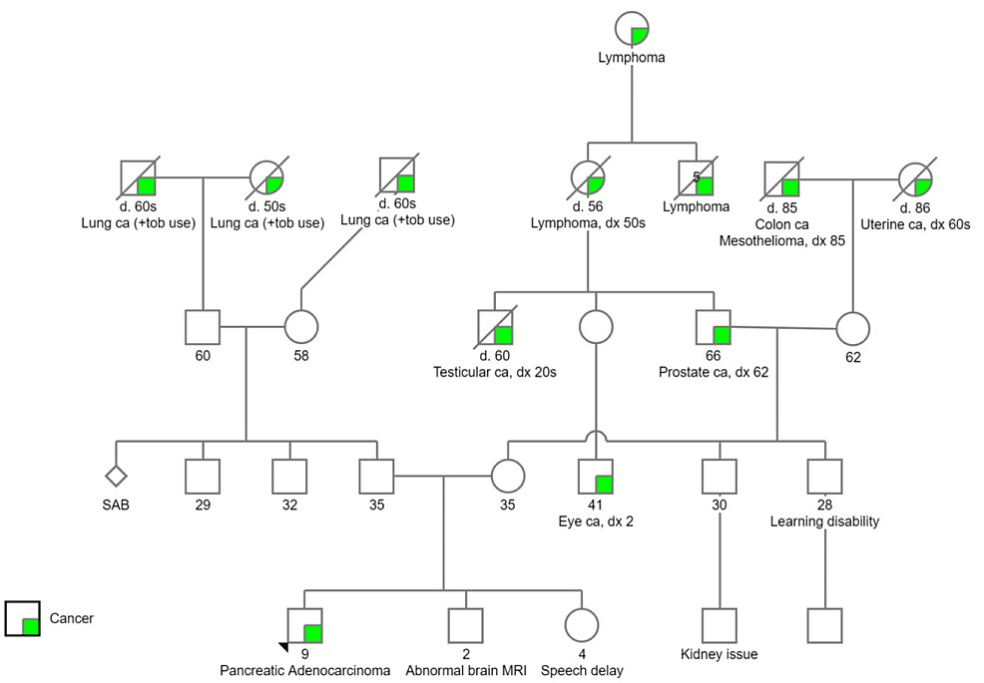
Family pedigree demonstrates four generations of individuals affected by cancer ca = cancer; tob = tobacco; d. = d. = age of death; dx = diagnosis; SAB = spontaneous abortion; MRI = magnetic resonance imaging

Given the diagnosis of pancreatic adenocarcinoma in a child, genetics was consulted for evaluation of an underlying cancer predisposition syndrome. The child’s family history is significant for cancer in both maternal and paternal relatives, however, no other relative had PDAC (Figure 4). Germline evaluation was conducted via commercial laboratory with a 29-gene pancreatic cancer panel, which revealed a c.345+6A>T variant of uncertain significance (VUS) in the *FANCC* gene. This VUS affects a nucleotide in the consensus splice site in intron 4.

## Discussion

PDAC is exceedingly rare in children. Malignant pancreatic tumors have an overall incidence of 0.46 cases per 1 million in individuals less than 30 years old, and of those, carcinomas represent <5% [8]. In the rare cases reported in adolescents, PDAC is most often identified in the late teenage years, with males tending to be more affected [9]. More than half of patients with PDAC present with distant metastases, and it is overall associated with a poor prognosis [10,11]. In adults, overall five-year survival is less than 10% [3,4]. In children, PDAC is associated with the worst prognosis of any childhood pancreatic malignancy [10,12]. Among seven children and adolescents diagnosed with PDAC in the SEER 21 database, the 15-year survival rate was 21% [9,11].

Overall, the prognosis improves in PDAC when tumors are localized to the pancreas and amenable to upfront surgical resection, though this unfortunately occurs only in a minority of patients (<20%) [1,13]. Patients such as the child we report here with metastatic disease are unlikely to benefit from upfront surgical resection of the primary tumor. Instead, patients with metastatic PDAC are treated with chemotherapy, with several regimens having shown survival benefits in clinical trials. In 2011, the PRODIGE trial demonstrated a significant benefit for median progression-free survival (6.4 vs. 3.3 months) and median overall survival (11.1 vs. 6.8 months) for patients treated with FOLFIRINOX compared to gemcitabine, the standard of care at the time [14,15]. In 2013, the MPACT trial demonstrated an overall survival benefit for albumin-bound paclitaxel (nab-paclitaxel) and gemcitabine in combination when compared with gemcitabine alone (8.7 months vs 6.6 months) [15,16]. Long-term survival was achieved for 3% of the patients from the nab-paclitaxel plus gemcitabine arm at 42 months, whereas no patients in the control arm survived at 42 months [15,17]. A retrospective comparative effectiveness study found that median overall survival was longer (9.27 months) for patients treated with FOLFIRINOX compared to nab-paclitaxel plus gemcitabine (6.87 months) [18].

With these survival benefits in mind, the NCCN recommends FOLFIRINOX or nab-paclitaxel plus gemcitabine both as evidence-based first-line treatment options for patients with PDAC with good performance status [15]. We therefore treated our patient with FOLFIRINOX initially, followed by a transition to gemcitabine plus nab-paclitaxel when he experienced tumor progression. Of note, the youngest patient included in the PRODIGE trial was 25 years old, with a median age of 61 [14]. Similarly, the youngest patient included in the MPACT trial was 27 years old, with a median age of 62 [16]. The study population is important to consider given the physiologic differences between pediatric and adult patients, especially when monitoring for adverse effects of chemotherapy. Our pediatric patient experienced only mild side effects of diarrhea, nausea and vomiting, cold sensitivity, and mild peripheral neuropathy during his treatment with FOLFIRNOX, none of which were dose-limiting toxicities. Side effects while receiving therapy with gemcitabine and nab-paclitaxel were similarly mild, consisting of fatigue, nausea, and vomiting. He did have a one-week delay in treatment secondary to thrombocytopenia.

PDAC has been reported to have a germline genetic association in about 5.5% of isolated cases, and 10-13% of familial or hereditary cohorts [6,7]. Several different hereditary syndromes have been implicated in PDAC, including Hereditary Breast and Ovarian Cancer (*BRCA1/BRCA2*), Familial Atypical Multiple Mole Melanoma (*CDKN2A*), Ataxia-Telangiectasia (*ATM*), Hereditary Pancreatitis (*PRSS1/SPINK1*), Peutz-Jeghers syndrome (*STK11/LKB1*), and Lynch syndrome (*MLH1/PMS2/MSH2/MSH6*) [6-8]. In addition, studies are linking new germline variants to PDAC annually, with numerous VUS in candidate genes being reported including *FANCC*, *CFTR*, *APC*, and *MUTYH* [6]. Emerging data has identified germline *FANCC* variants as enriched in patients with PDAC [19,20], and our patient was found to have an intronic VUS in the *FANCC* gene. Additional studies are needed to allow for variant reclassification to better determine the potential involvement of *FANCC* variants in the pathogenesis of PDAC as it applies to this case and others.

## Conclusions

Here, we present a rare case of pancreatic adenocarcinoma in a nine-year-old child. Very few cases of PDAC have been reported in pediatric patients in the literature, and cases in patients under 10 years old are essentially unheard of. At this time, the care of a pediatric patient with PDAC involves extrapolation of adult guidelines for preferred treatment options. In addition, genetic testing should be considered for any pediatric patient presenting with PDAC. This patient had VUS in the *FANCC* gene, which is one of several candidate genes with emerging associations with PDAC. Further studies are important to allow for variant reclassification for *FANCC* as it relates to PDAC, which would have important implications with regard to screening guidelines and chemotherapeutic opportunities.
